# Shape Discrimination of Individual Aerosol Particles Using Light Scattering

**DOI:** 10.3390/s23125464

**Published:** 2023-06-09

**Authors:** Yan Han, Lei Ding, Yingping Wang, Haiyang Zheng, Li Fang

**Affiliations:** 1 Anhui Institute of Optics and Fine Mechanics, Hefei Institutes of Physical Sciences, Chinese Academy of Sciences, Hefei 230031, China; 2 Graduate School of Science Island Branch, University of Science and Technology of China, Hefei 230026, China

**Keywords:** individual aerosol particle, shape of particulate matter, polarized light scattering, angle-resolved light scattering, aerodynamic particle size

## Abstract

We established an experimental apparatus by combining polarized light scattering and angle-resolved light scattering measurement technology to rapidly identify the shape of an individual aerosol particle. The experimental data of scattered light of Oleic acid, rod-shaped Silicon dioxide, and other particles with typical shape characteristics were analyzed statistically. To better study the relationship between the shape of particles and the properties of scattered light, the partial least squares discriminant analysis (PLS-DA) method was used to analyze the scattered light of aerosol samples based on the size screening of particles, and the shape recognition and classification method of the individual aerosol particle was established based on the analysis of the spectral data after nonlinear processing and grouping by particle size with the area under the receiver operating characteristic curve (AUC) as reference. The experimental results show that the proposed classification method has a good discrimination ability for spherical, rod-shaped, and other non-spherical particles, which can provide more information for atmospheric aerosol measurement, and has application value for traceability and exposure hazard assessment of aerosol particles.

## 1. Introduction

In aerosol particle diagnostic techniques, traditional methods of aerosol particle assessment often include microscopic inspection or molecular analysis of filters, suffering from low time resolution due to relatively long analytical procedures, and may cause damage to particles. Optical methods are widely used in monitoring ambient air pollution, cloud microphysics, aerosol particle detection in the workplace, and detection of bioaerosol release since they have the advantages of non-destructive and fast response when compared to traditional detection methods [1,2,3,4]. The optical properties of atmospheric aerosol are related to their size, shape, concentration, and chemical composition [5,6]. The size and shape of airborne particles determine their behavior in the air, including generation, transmission, and sedimentation. In many circumstances, the shape of particles can give some indication of the source of those particles and hence facilitate more effective monitoring or contamination control [7]. Among all optical measurement methods, elastic light scattering provides the strongest signal, far more than inelastic light scattering, which facilitates detection. Therefore, aerosol particle elastic scattering technology has been widely used in recent years, especially for single particles [8,9].

Many researchers have studied the scattering properties of particles through computational simulations [10,11,12,13]. In the experimental study of the scattered light of aerosol particles, the research on the angle-resolved light scattering of particles was started earlier [14,15,16]. Then, various detection techniques for scattered light distribution have been further developed. Kaye et al. have collected scattered light with an ellipsoid mirror and detected high-resolution scattering patterns with a wide range of solid angles using an intensified charge-coupled-device camera and compared them with theoretical scattering patterns [17]. After that, they developed a real-time rapid monitoring system in the early stage for detecting the shape and size characteristics of airborne particles [7]. Hirst et al. designed an aircraft-mounted probe to provide in situ data on cloud particle shape, size, and concentration to help distinguish super-cooled water droplets and ice crystals in 1–25 μm mixed-phase clouds [18], some other researchers have also designed related devices and conducted experiments [4,5,19]. With the further development of light scattering technology in the detection of aerosol particles, the polarization characteristics of aerosol particles have been applied in climate research, atmospheric and oceanic environmental monitoring, astrophysics, bioaerosol science, and other fields [20,21,22,23]. A method based on polarized light scattering was proposed by Wang et al. to identify different kinds of suspended particles rapidly and massively [24]. Li et al. present an in situ online method to characterize aerosols by synchronous parallel polarization scattering analysis with a multi-angle polarization scattering instrument [25]. As the scattered light contains abundant information about particle shape, size, and other properties, which will bring difficulties to the analysis. Therefore, there are still some challenges in rapidly classifying or even identifying the types of particles through the information contained in the scattered light of aerosol particles.

To rapidly discriminate the shape of the individual aerosol particles, we proposed an experimental setup capable of simultaneously measuring the intensity of angle-resolved light scattering, polarization characteristics, and aerodynamic diameter of particles in this paper. Based on the size screening of aerosol particles with different shapes, the scattered light of particles getting under two wavelengths was analyzed by Partial Least Squares Discriminant Analysis (PLS-DA) method, and a prediction model between the scattered light and shape of individual particles under experimental conditions was established. The preliminary results illustrate the capability of this method to distinguish the spherical, rod-shaped, and other non-spherical particles.

## 2. Experimental Methods

### 2.1. Experimental Setup

**Figure 1:**
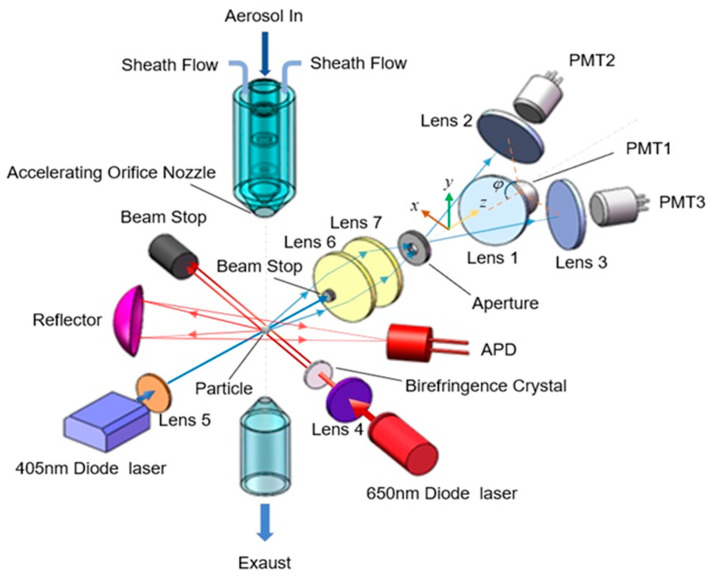
Schematic diagram of the experimental setup.

Figure 1 is a schematic diagram of the polarization and angular resolution light scattering measuring device for individual aerosol particles. Firstly, various polydisperse aerosol particles with different shape characteristics were pumped into the aerosol injection detection unit. During the sample injection, the aerosol particles in the sample airflow were collimated and accelerated, and then the target particles enter the scattering light detection area. In the optical measurement area, aerosol particles were illuminated by two partially overlapping laser beams first, which focused under the particle stream and close to the inlet nozzle. The linearly polarized light with a wavelength of 650 nm was emitted by a diode laser, and the vertical and horizontal components of the beam were split into two separate orthogonally polarized beams with an interval of about 100 μm by using a birefringent plate (YVO4 orthotropic yttrium vanadate crystal). The light scattered by the particle was collected by a reflector which was placed at 45° forward to the laser axis and focused onto an avalanche photodiode (APD) detector. The detector then converts the light pulses into electrical pulses generating a double-crested signal of scattered light corresponding to horizontal and vertical polarized light, respectively. The time between the crests is called the time-of-flight (TOF1), it could provide aerodynamic particle-size information [26]. Then the particles pass through a light beam with a wavelength of 405 nm, which is located below the 650 nm laser beam at a distance of less than 1 mm, and the corresponding forward elastic scattering light generated by the particle was collimated and aggregated by Lens6 and Lens7, respectively. Among them, Lens6 has an extinction device in the center to block the incident laser light (so the receiving range of the polar angle of the scattered light is 6°~18°), and Lens 7 focuses the scattered light through the aperture to reduce the effect of stray light. Lens 7 focuses the scattered light through the aperture to reduce the effect of stray light. Finally, the scattered light is concentrated again by three lenses (which were symmetrically distributed on the forward space with azimuth interval 120°) and received by the photomultiplier tubes (PMTs) located at the focus of the lenses, respectively.

**Figure 2:**
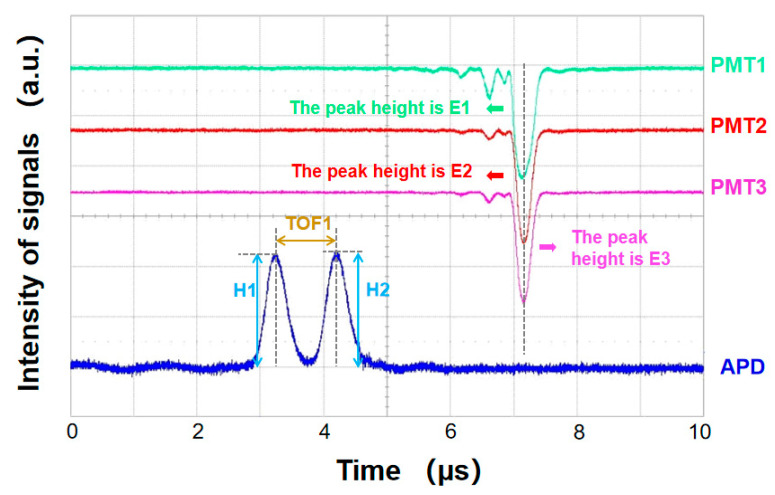
Schematic diagram of scattering spectrum signal of single aerosol particle collected by oscilloscope.

A digital oscilloscope (WaveRunner104Xi-A, LeCroy (Chestnut Ridge, NY, USA)) was used to display and store the amplified signal from the APD and PMTs for later data processing. The sampling interval of the oscilloscope was set to 1 ns. The typical signals of a single oleic acid particle displayed on the oscilloscope are shown in Figure 2. As previously mentioned, TOF1 is the time-of-flight of the particle passing through two separate orthogonally polarized beams (usually a few hundred nanoseconds to a few microseconds), and proportional to the aerodynamic diameter. H1 and H2 correspond to the peak values of the bimodal signal produced by APD when particles pass through horizontally polarized incident light and vertically polarized incident light, respectively. E1, E2, and E3 correspond to the peak values of the single-peak signals produced by the three PMT when the particles pass through the laser with a wavelength of 405 nm, respectively.

### 2.2. Calculation Method of Scattered Light

**Figure 3:**
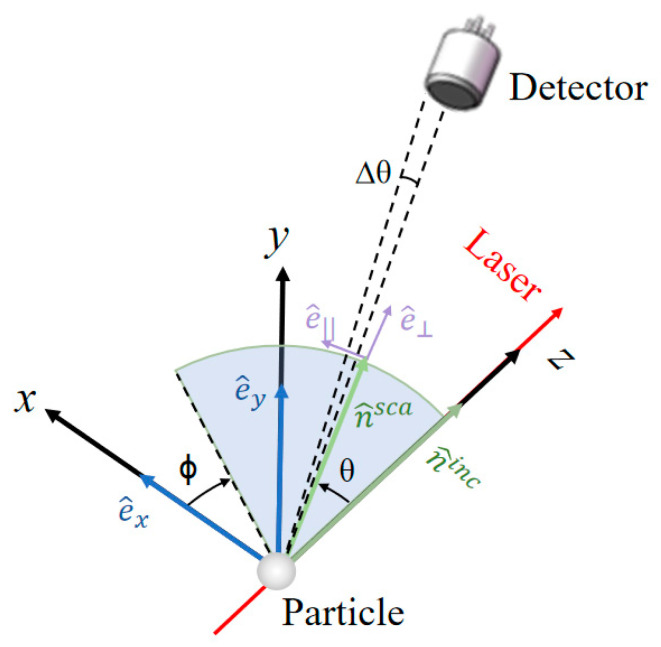
Schematic diagram of light scattering coordinate system.

In the laboratory coordinate system $L\left(x,y,z\right)$, the directions of the incident light and scattering light involved in the light scattering process of the particle are shown in Figure 3. The incident light is generally set to propagate along the positive direction of the *z*-axis of the laboratory coordinate system, which means the scattering angle $\theta^{inc}$ and azimuthal angle $\phi^{inc}$ of the incident light are both equal to 0, and the incident light of arbitrary polarization can be decomposed into two orthogonal electric vectors ${\hat{e}}_{x}$ and ${\hat{e}}_{y}$. The plane formed by the directions of the scattered beam ${\hat{n}}^{sca}$ and the incident beam ${\hat{n}}^{inc}$ is called the scattering plane. The scattering light can be decomposed into an electric vector ${\hat{e}}_{∥}$ parallel to the plane and an electric vector ${\hat{e}}_{⊥}$ perpendicular to the plane.

Combined with the T-matrix theory and Muller matrix, the calculation method of the differential scattering cross-section of particles under incident light with different polarization characteristics can be derived, and the specific derivation process has been explained in another article [27]. When the incident light is linearly polarized along the *x*-axis, linearly polarized along the *y*-axis, or non-polarized, then the calculation method of corresponding differential scattering cross-section $\frac{dC^{sca}}{d\Omega }$ can be calculated by Equation (1), Equation (2), or Equation (3), respectively.

(1) $$\frac{dC_{x}^{sca}}{d\Omega }=Z_{11}+Z_{12},$$

(2) $$\frac{dC_{y}^{sca}}{d\Omega }=Z_{11}-Z_{12},$$

(3) $$\frac{dC_{n}^{sca}}{d\Omega }=Z_{11},$$

where $Z_{11}$ and $Z_{12}$ are the elements of the Mueller matrix for scattering by individual particle. In addition, since the detector has a certain angle of view, the scattering light in the detection area needs to be calculated integrally.

(4) $$I^{sca}=I^{inc}\int_{\varphi_{0}-\Delta \phi }^{\varphi_{0}+\Delta \phi }\int_{\theta_{0}-\Delta \theta }^{\theta_{0}+\Delta \theta }\frac{dC^{sca}}{d\Omega }(\theta ,\phi )\sin\theta d\theta d\phi .$$

The $\theta_{0}$ and $\phi_{0}$ in the above equation are the scattering angle and azimuth of the detector’s center position, Δθ is half of the angle of view of the detection receiving surface (as shown in Figure 3), and Δϕ is the integral range of the azimuthal angle. If the receiving surface of the scattered light is circular, and the normal direction of the center passes through the interaction point (origin) of the laser and the particle, then Δϕ can be expressed as [28]:

(5) $$\Delta \phi =\cos^{-1}\left(\frac{\cos\Delta \theta -\cos\theta_{0}\cos\theta }{\sin\theta_{0}\sin\theta }\right).$$

### 2.3. Sample Generation

The polydisperse aerosol particles used in the experiment can be generated by the self-made polydisperse aerosol generator NAG2210, which works in a similar way to the atomizer. Firstly, the sample should be prepared into solution by selecting a suitable solvent, then a certain volume of the prepared solution should be measured and put in a container. After that, the high-speed airflow formed by compressed air passing through the small nozzle was used to drive the solution in the container to form a liquid jet. The generated liquid jet will hit the inner wall of the container at a high speed, split into liquid droplets and splash to the surrounding area, forming mist-like particles, then enter the buffer chamber with the airflow. In the buffer chamber, when the volatile solvent in the droplet evaporated, the formed target aerosol particles will be output from the buffer chamber with the airflow and enter the measuring device. For liquid solutions (such as Oleic acid solution), aerosol droplets with different size characteristics can be formed according to different concentrations of solutions. For the solution containing insoluble solid particles, the size distribution of aerosol particles depends on the solid particles themselves. The monodisperse aerosols were generated by a flow-focusing monodisperse aerosol generator (FMAG1520, TSI).

**Figure 4:**
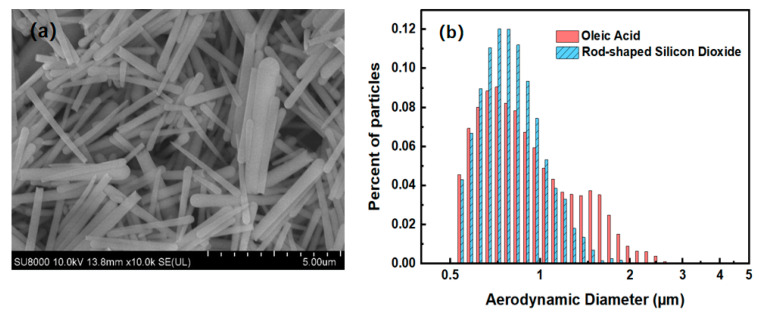
(**a**) Electron microscope image of rod-shaped Silicon dioxide particles synthesized by reversed-phase microemulsion method. (**b**) Size distribution of aerosol samples.

Two aerosol samples with different shapes were studied experimentally, including Oleic acid particles and rod-shaped Silicon dioxide particles. Since it is difficult to obtain rod-like aerosol samples, so we synthesized rod-shaped Silicon dioxide particles by ourselves using the reversed-phase microemulsion method [29], and the scanning electron microscope (SEM) image of the sample is shown in Figure 4a. It can be seen that almost all the synthesized Silicon dioxide particles are rod-shaped. Oleic acid particles cannot be photographed by electron microscope because of their properties, but according to relevant literature, the shape of Oleic acid particles is considered spherical [7,30]. Both Oleic acid and Silicon dioxide have a refractive index of 1.46 [29,31]. The size distribution of the four aerosol particles was measured by Aerodynamic Particle Sizer Spectrometer (APS3321), respectively. To better compare the size distribution of different aerosol particles, the frequency distribution diagram of the particle size distribution of each sample was drawn, and the results are shown in Figure 4b. As can be seen from Figure 4b, the aerodynamic particle diameter *D_a_* of two aerosol particles both within 4 μm, and the size distribution of Oleic acid particles is slightly wider than that of rod-shaped Silicon dioxide particles.

## 3. Results and Discussion

### 3.1. Extraction and Correction of the Spectral Signal

#### 3.1.1. Signal Extraction

**Figure 5:**
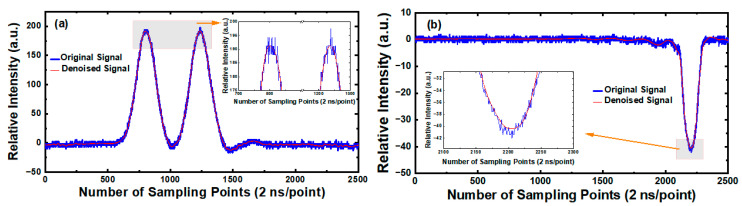
Schematic diagrams and local enlarged schematic diagrams of original signal and denoised signal of single Oleic acid particle. (**a**) The spectral signal corresponds to APD. (**b**) The spectral signal corresponds to PMT.

Due to the influence of light and electrical noise during the experiment, the shape of the real spectral signal will be distorted, and the signal-to-noise ratio will be decreased. Therefore, in the processing of spectral signals, noise reduction is often needed. Wavelet soft threshold denoising method was applied to preprocess the scattered light signals detected by APD and PMTs, respectively. Taking Oleic acid particles as an example, Figure 5 shows the comparison results between the original spectral signals of Oleic acid particles output by APD and PMTs and their corresponding signals after noise reduction. Figure 5a is the spectral signal corresponding to the APD, and Figure 5b is the spectral signal corresponding to the PMT. The blue solid line in the figure represents the original spectral signal before denoising, while the red solid line represents the spectral signal after denoising. It can be seen that, compared with the original spectral signal, the noise level of the spectral signals after denoising is obviously reduced, and the smoothness of the signals is significantly improved.

Wavelet denoising was carried out on the original spectral data of all samples collected by the oscilloscope, and the information of peak position and peak value are extracted, respectively based on the denoised scattering light signals. For each aerosol particle, six optical parameters (TOF1, E1, E2, E3, H1, and H2) to be analyzed can be obtained after proper processing of spectral information obtained by peak seeking. The meaning of each optical parameter has been explained in Section 2.1.

**Figure 6:**
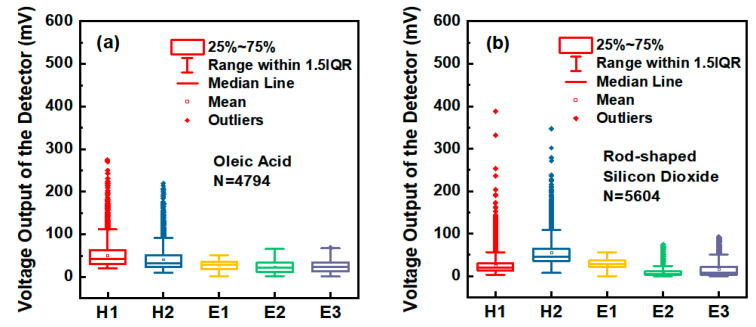
The intensity distribution of scattered light from aerosol samples. (**a**) Oleic acid aerosol particles. (**b**) Rod-shaped Silicon dioxide aerosol particles.

The intensity distribution of scattered light of each sample measured by APD and PMTs in the experiment is shown in Figure 6. The abscissa in the figure represents scattered light signals received by each detector during the light-scattering process of individual particles, and the ordinate represents the output voltage of the oscilloscope corresponding to each signal. Figure 6a,b represents Oleic acid aerosol particles, and rod-shaped Silicon dioxide aerosol particles, respectively. As can be seen from Figure 6, the intensity distribution range of scattered light received by the PMTs corresponding to thousands of (N = 4794) Oleic acid particles is basically the same, and the intensity distribution of the two peaks of the double-crested signal received by the APD is similar as well, but the intensity of H2 is slightly less than H1 on the whole. However, for thousands of (N = 5604) rod-shaped Silicon dioxide particles, the intensity distribution of the scattered light received by each detector is obviously different from that of the Oleic acid particles, in which the intensity of H2 is significantly higher than that of H1, and the intensity of E1 is significantly higher than that of E2 and E3, indicating that the intensity of scattered light from aerosol particles with different shapes received by each photoelectric detector has different distribution characteristics.

#### 3.1.2. Correction of Light Intensity

**Figure 7:**
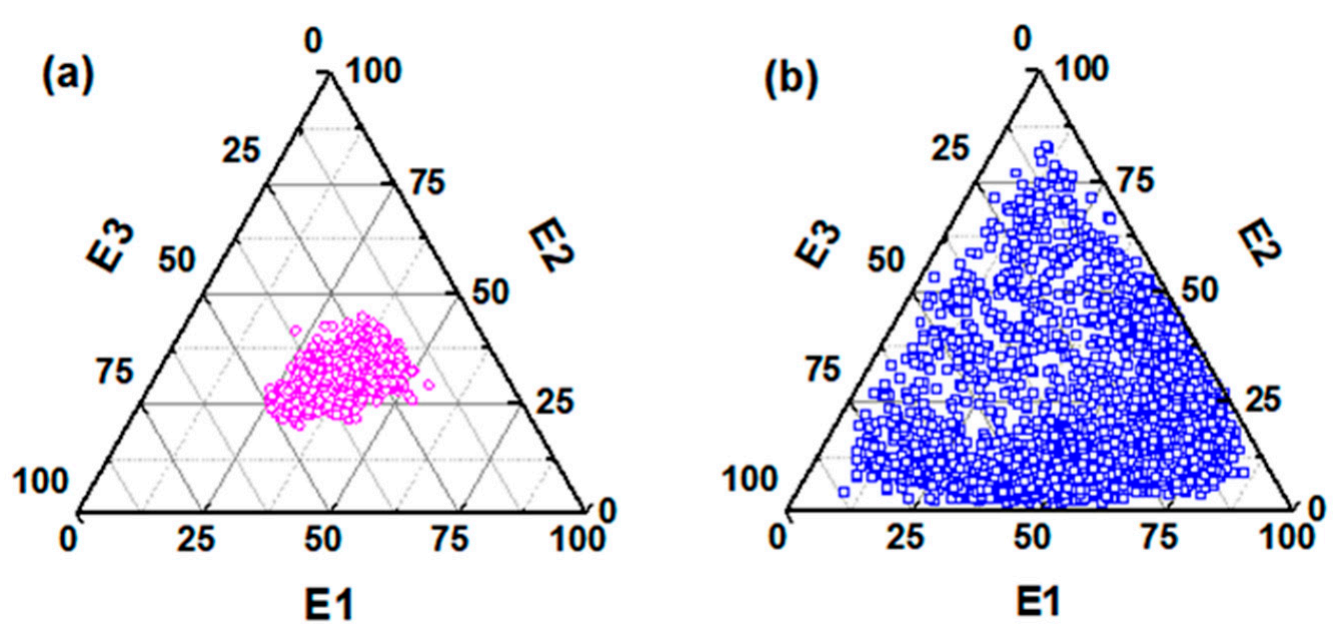
Relative sizes of corrected scattered light intensity E1, E2, and E3 of two aerosol particles. (**a**) Oleic acid aerosol particles. (**b**) Rod-shaped Silicon dioxide aerosol particles.

To avoid the influence of the difference in the photoelectric conversion efficiency of the three PMTs on the measured results of the intensity of scattered light, spherical Oleic acid particles were used to modify the response intensity of each PMT. Figure 7 shows the relative magnitude of scattered light intensity E1, E2, and E3 of each aerosol particle in different spatial orientations after third-order polynomial correction. It can be seen that the Oleic acid aerosol particles in Figure 7a are concentrated in the central region of the ternary phase diagram, which indicates that the intensity of scattered light from an individual Oleic acid particle in three spatial directions is relatively close. The rod-shaped Silicon dioxide aerosol particles in Figure 7b tend to be distributed in the lower right corner of the ternary phase diagram, indicating that the intensity of scattered light of most rod-shaped Silicon dioxide particles at PMT1 position (corresponding horizontal direction in spatial orientation) is greater than that at PMT2 and PMT3 positions. The results also prove that the orientation of the rod-shaped particles tends to be parallel to the airflow in the injection flow, which is consistent with the conclusion of Hirst et al. [32].

### 3.2. Screen the Time-of-Flight

**Figure 8:**
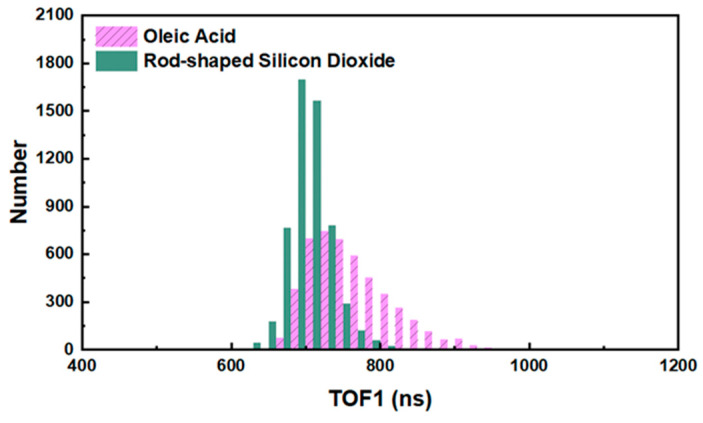
The distribution of time-of-flight of Oleic acid particles and rod-shaped Silicon dioxide particles.

**Figure 9:**
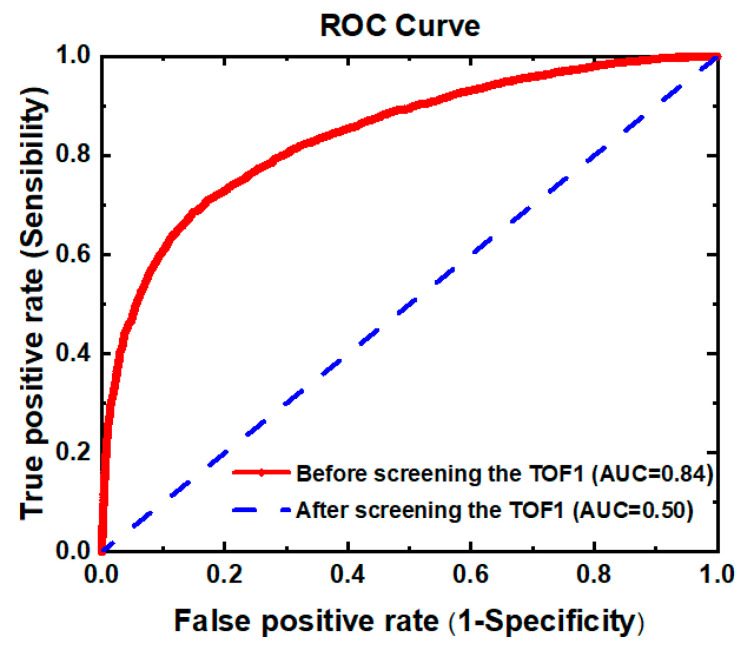
The AUC of Oleic acid particles and rod-shaped Silicon dioxide particles based on their time-of-flight.

Since the distribution range of time-of-flight (corresponds to the size of the particle) of Oleic acid aerosol particles in the experiment is not completely consistent with that of rod-shaped Silicon dioxide aerosol particles (as shown in Figure 8), it is necessary to confirm whether the size of particles will affect the analysis and identification of the two kinds of aerosol particles. To evaluate the performance of the discrimination between Oleic acid aerosol particles and rod-shaped Silicon dioxide aerosol particles based on their time-of-flight, the receiver operating characteristic (ROC) curves were applied here to analyze the two kinds of particles, and the results are shown in Figure 9. ROC curve can be used to evaluate the performance of the classifier. Generally speaking, the closer the value of the area under the ROC curve (AUC) is to one, the better the classification effect of the method. It can be seen that the AUC corresponding to the red line in Figure 9 is 0.84, which indicates that the time-of-flight of the two kinds of aerosol particles has a certain ability to distinguish their categories. Therefore, to eliminate the influence of particle size on the classification of Oleic acid and rod-shaped Silicon dioxide particles and the intensity of their scattered light, we screened the spectral data to be analyzed according to their time-of-flight. The spectral data of the two kinds of aerosol particles which have the same TOF1 are selected and stored as matrix X0 and X1, respectively (both X0 and X1 are matrices of order 2335 × 6, and 2335 is the number of target particles screened out from the two samples, respectively). The ROC curve of Oleic acid particles and rod-shaped Silicon dioxide particles was drawn by taking the time-of-flight contained in the matrix X0 and X1 as the criterion, and the results are shown in the blue line in Figure 9. The value of AUC corresponding to the blue line in Figure 9 is 0.50, indicating that the aerosol particles of Oleic acid and rod-shaped Silicon dioxide could not be distinguished by TOF1 alone.

### 3.3. Modeling and Analysis

By standardizing the spectral data contained in the matrix X0 and X1, respectively, the corresponding data matrix to be analyzed can be obtained, which contains six variables. To clarify the relationship between the above six variables and particle shape, the standardized spectral data of scattered light of aerosol particles were discriminated and analyzed with six variables as independent variables and aerosol particle shape as response variables. Among the multivariate discriminant analysis methods, principal component analysis (PCA), and partial least squares discriminant analysis (PLS-DA) are commonly used. The above two methods are based on principal component regression and partial least squares regression, respectively to reduce the dimension of data, establish models, and conduct discriminant analysis on the prediction results. Both two methods can effectively analyze and process high-dimensional data, but the PLS-DA is a supervised discriminant analysis, which can effectively reduce the influence of multiple correlations between variables. Therefore, the PLS-DA method was used in this paper to analyze the spectral data of scattered light from various aerosol particles.

**Figure 10:**
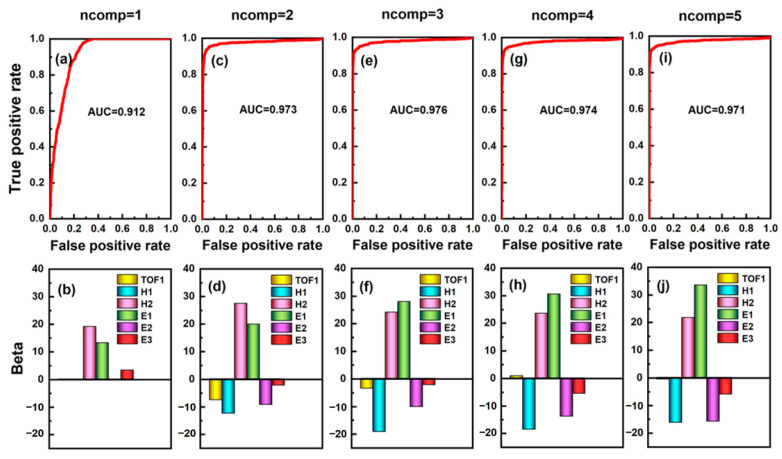
The results of the AUC of the classification model and the Beta coefficient correspond to the respective variables when PLS-DA extracted different number of principal components. (**a**,**b**) ncomp = 1. (**c**,**d**) ncomp = 2. (**e**,**f**) ncomp = 3. (**g**,**h**) ncomp = 4. (**i**,**j**) ncomp = 5.

Since in partial least squares analysis, the number of extracted principal components will affect the prediction results of the model, it is necessary to set the appropriate number of principal components according to the specific situation. Generally speaking, if the number of independent variables is *n*, the maximum number of principal components extracted for dimensionality reduction can be set to *n* − 1. Therefore, the number of extracted principal components was set as one to five, respectively, and the ROC curves of Oleic acid particles and rod-shaped Silicon Dioxide were drawn based on the predicted values of the corresponding models when different numbers of principal components were extracted. The dependent variables of the model corresponding to Oleic acid and rod-shaped Silicon Dioxide were set as 0 and 100, respectively, and the results were shown in Figure 10. The top row of pictures in Figure 10 shows the corresponding results of the ROC curves, while the bottom row of pictures shows the Beta coefficients of the respective variables corresponding to the extraction of different numbers of principal components. It can be seen that when the number of principal components extracted is different, the AUC has different results, and the values of Beta coefficients corresponding to each independent variable are also quite different. When the number of extracted principal components was three, the maximum value of AUC can be obtained, which is 0.976. Meanwhile, it can be seen from Figure 10f that the independent variables *H*1, *H*2, and *E*1 have a relatively great influence on the model under this condition.

Table 1 shows the distribution of the explainable percentage of variance (PCTVAR) for the variables when different numbers of principal components were extracted. It can be seen that when the number of principal components was three, the explanatory ability of the established model for independent variables and dependent variables was 0.7575 and 0.6759, respectively. Since only linear combination processing was carried out on the independent variables in the PLS-DA at this time, nonlinear preprocessing of six independent variables was considered in order to further improve the explanatory ability of the model.

To perform nonlinear preprocessing for the above independent variables, based on previous research experience and the characteristics of light scattered by aerosol particles in experiments, we introduce the following two parameters in the nonlinear processing of the independent variable: *AP_f_* and *AF_f_*. The two parameters are similar to the asymmetry factor (*A_f_*) proposed by Kaye et al. [7], and can be expressed as follows:

(6) $$AP_{f}=\left|\frac{H1-H2}{H1+H2}\right|\times 100,$$

(7) $$AF_{f}=\left|\frac{E1-Mean(E2,E3)}{Max(E1,E2,E3)+Min(E1,E2,E3)}\right|\times 100.$$

By using Equations (6) and (7), two matrices of 2335 × 3 order can be obtained, and the three columns of data correspond to *TOF*1, *AP_f_*, and *AF_f_*, respectively. The PLS-DA was carried out on the transformed spectral data, and the number of extracted principal components was set as one and two, respectively. The results are shown in Table 2. As can be seen from Table 2, after the nonlinear processing of the six independent variables, the ability of the prediction model to distinguish the shape of particulate matter and explain variables has been significantly improved. When the number of principal components was set to two, the corresponding result is better than that when the number of extracted principal components was set to one.

When the number of extracted principal components was set to two, the value of AUC is 0.9828, and the expression of the corresponding prediction model is shown in Equation (8), where *F_s_* represents the predicted value of the model. Because the values of *TOF*1 of the aerosol samples detected in the experiment are all within 10 after central standardization, and the values of *AP_f_* and *AF_f_* range from 0 to 100 according to their definitions by combining the coefficients of each variable in Equation (8), it can be seen that the value of the first term is about two to three orders of magnitude smaller than the value of the second and third terms in this prediction model, indicating that the influence of *TOF*1 is very small and almost negligible compared to *AP_f_* and *AF_f_*. Therefore, to ensure that the predicted values of the models corresponding to different aerosol particles have the same range, the *TOF*1 variable is omitted for re-modeling, and the result is shown in Equation (9). By comparing Equation (8) with Equation (9), it can be seen that the difference between the coefficients of *AP_f_* and *AF_f_* in the two equations is extremely small. According to Equation (9), the value of *F_s_* ranges from −9.9722 to 203.8778. To conveniently identify and classify the shape of aerosol particles according to the value of *F_s_*, a simple mathematical transformation was carried out on Equation (9), and its value range was adjusted to 0~100. The transformed prediction model is shown in Equation (10).

(8) $$F_{s}=0.0192\times TOF1+0.8141\times AP_{f}+1.3260\times AF_{f}-10.0097,$$

(9) $$F_{s}=0.8111\times AP_{f}+1.3274\times AF_{f}-9.9722,$$

(10) $$F_{s}=0.379\times AP_{f}+0.621\times AF_{f}.$$

**Figure 11:**
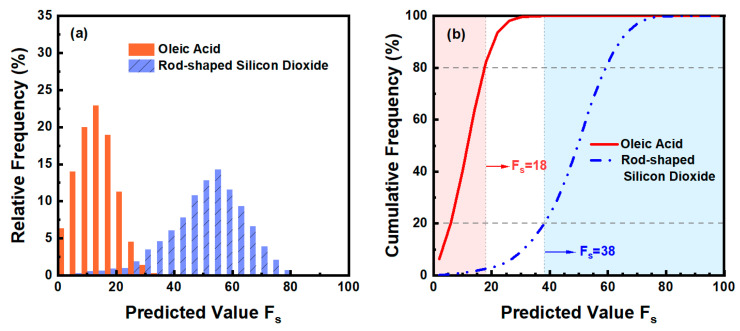
The distribution of *F_s_* of Oleic acid aerosol particles and rod-shaped Silicon dioxide aerosol particles. (**a**) Relative frequency. (**b**) Cumulative frequency.

By applying Equation (10) to all aerosol particles of polydispersed Oleic acid and rod-shaped Silicon dioxide, the distributions of predicted values corresponding to the two aerosol particles can be obtained, as shown in Figure 11. The histogram in Figure 11a shows the relative frequency distribution of predicted values *F_s_* corresponding to various aerosol samples (the interval between adjacent intervals is 4). It can be seen that the value of *F_s_* of spherical Oleic acid aerosol particles is generally small, and the value of *F_s_* at the highest point of its relative frequency histogram is 14. For rod-shaped Silicon dioxide aerosol particles, the value of *F_s_* is obviously higher than that of the Oleic acid aerosol particles, and the overall distribution is closer to the right side of the axis. The highest point of relative frequency histogram appears at *F_s_* is 54. Figure 11b represents the distribution trend of the cumulative frequency of the predicted value *F_s_* corresponding to the above aerosol samples. It can be seen that there is a significant difference between the distribution trend of cumulative frequency of Oleic acid aerosol particles and rod-shaped Silicon dioxide aerosol particles. Considering that some particles with unsatisfactory shapes may be produced during the preparation and generation, which will affect the shape classification of particulate matter, therefore, the values of *F_s_* of Oleic acid particles corresponding to the cumulative frequency of 80% and rod-shaped Silicon dioxide particles corresponding to the cumulative frequency of 20% can be considered as the distinguishing thresholds of spherical aerosol and rod-shaped aerosol, respectively (corresponding to *F_s_* = 18 and *F_s_* = 38, respectively). Because the abscissa in Figure 11 is the center value of each interval, the criteria for judging spherical aerosol particles and rod aerosol particles should be *F_s_* < 20 and *F_s_* > 36, respectively. When the value of *F_s_* of the particle is between 20 and 36, it can be considered as other non-spherical particles.

### 3.4. Group by Particle Size

Since the polarized light scattering method of a single particle will affect by particle size [28], to explore the influence of light scattering parameters on the discrimination of aerosol particle shape in different particle size ranges, the Oleic acid aerosol particles and rod-shaped Silicon dioxide aerosol particles were grouped according to particle size based on the screening of time-of-flight, and the spectrum data in each particle size range were analyzed by PLS-DA, respectively.

**Figure 12:**
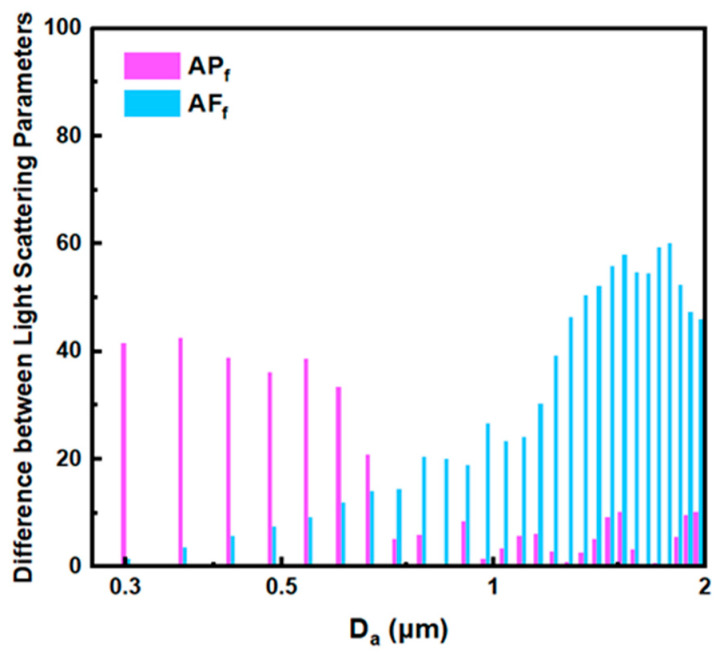
Difference of light-scattering parameters between Oleic acid and rod-shaped Silicon dioxide particles under different aerodynamic particle sizes.

Taking spherical Oleic acid particles and ellipsoidal Silicon dioxide particles with an aspect ratio of 3:1 as examples, the difference in light scattering parameters of the two particles under different aerodynamic particle sizes was calculated by simulation and interpolation method (the orientation of the elongated particles was set parallel to the airflow direction), and the results are shown in Figure 12. It can be seen that at the smaller particle size range, the difference in *AP_f_* between the two aerosol particles is large, while the difference in *AF_f_* is small. With the increase of aerodynamic particle size, the difference in *AF_f_* between the two aerosol particles increases gradually, while the difference in *AP_f_* decreases significantly.

**Figure 13:**
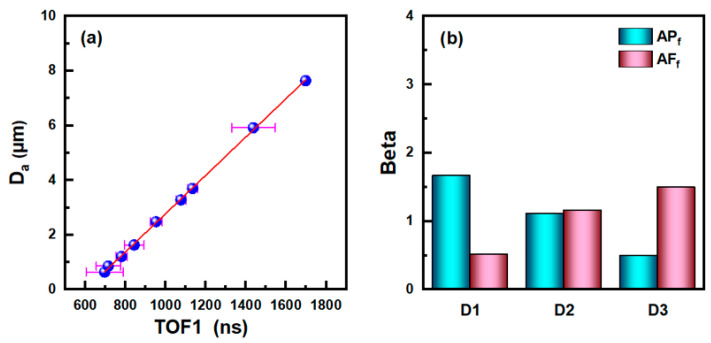
(**a**) Conversion relationship between time-of-flight of aerosol particles and aerodynamic diameter. (**b**) Beta coefficients of *AP_f_* variable and *AF_f_* variable of the models in different particle size segments.

Due to the time-of-flight of the aerosol particles measured in the experiment can only be converted into the aerodynamic diameter *D_a_* through the corresponding conversion equation, therefore, several monodisperse aerosol particles generated by the monodisperse aerosol generator FMAG1520 were used to calibrate the experimental device, and the results are shown in Figure 13a. The abscissa in Figure 13a represents the time-of-flight of the particle, and the ordinate represents its aerodynamic diameter. By referring to the fitting results of the calibration curve of the APS3321 device, the conversion equation between time-of-flight and aerodynamic diameter of the experimental device can be expressed as Equation (11):

(11) $$D_{a}=\frac{584.80812-109.6145x^{0.5}+3.4974x}{1+9.09183x^{0.5}-0.06727x}.$$

Based on the calculation results in Figure 12, the polydisperse Oleic acid droplets and rod-shaped Silicon dioxide particles were roughly divided into three subgroups according to their aerodynamic diameter, including the D1 (<0.54 μm), D2 (0.54~0.82 μm), and D3 (>0.82 μm). PLS-DA was performed on the spectral data of Oleic acid droplets and rod-shaped Silicon dioxide particles in the three particle size ranges, respectively, and the corresponding model evaluation indexes are shown in Table 3. It can be seen that the AUC of each particle size segment are 0.9950, 0.9905, and 0.9787, respectively, which indicates that the corresponding prediction model has a good discrimination effect on Oleic acid droplets and rod-shaped Silicon dioxide particles. In addition, the models also have a good ability to explain independent variables and dependent variables, in which the PCTVAR of independent variables in each particle size segment are 0.9991, 0.9990, and 0.9856, respectively, and the PCTVAR of dependent variables are 0.8515, 0.7938 and 0.7029, respectively.

Figure 13b shows the values of Beta coefficients corresponding to *AP_f_* and *AF_f_* variables in the model of each particle size segment. It can be seen that the relative values of Beta coefficients of the two variables are different in three particle size segments, and *AP_f_* has a greater influence on the model in the smaller particle size segment (D1), which shows that *AP_f_* has a better shape discrimination ability than *AF_f_* for particles in D1 segment. With the increase of particle size, the Beta coefficient of *AP_f_* decreases gradually, while the Beta coefficient of *AF_f_* shows an increasing trend. It shows that *AF_f_* is better than *AP_f_* in distinguishing the shape of aerosol particles in the larger particle size segment (D3) while the influence of the two parameters on the model is roughly equal in the D2 segment. In addition, it can be seen from Figure 13b that the results obtained by PLS-DA on the spectral data of aerosol particles are in good agreement with the calculated results under experimental conditions.

Similarly, to divide the aerosol particle shape within each particle size segment conveniently according to the *F_s_*, the range of predicted values of each model was adjusted to 0~100 by Equations (12)–(14). Referring to the method of threshold selection in Section 3.3, the values of *F_s_* of Oleic acid droplets corresponding to the cumulative frequency of 80% and rod-shaped Silicon dioxide particles corresponding to the cumulative frequency of 20% were used as the thresholds for separating spherical aerosols from rod-shaped aerosols in each particle size segment.

(12) $$F_{s}^{D1}=0.7642\times AP_{f}+0.2358\times AF_{f},$$

(13) $$F_{s}^{D2}=0.4898\times AP_{f}+0.5102\times AF_{f},$$

(14) $$F_{s}^{D3}=0.2505\times AP_{f}+0.7495\times AF_{f}.$$

### 3.5. Preliminary Laboratory Validation

**Figure 14:**
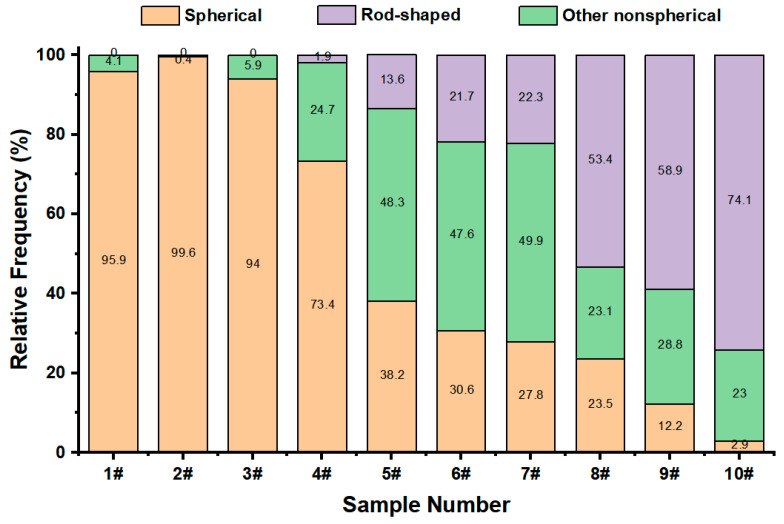
Identification and classification results of various aerosol samples with different shape characteristics.

To verify the effectiveness of the discrimination method proposed in Section 3.4, a variety of aerosol samples with different shape characteristics were used for the classification test, and the results are shown in Figure 14. The abscissa in Figure 14 represents the corresponding numbers of various aerosol samples, while the ordinate represents the proportion of spherical particles, rod-shaped particles, and other non-spherical particles in various aerosol samples. Among them, samples 1# and 2# are monodisperse Oleic acid droplets with a median aerodynamic diameter of 2.18 μm and 3.22 μm, respectively. Sample 3# is monodisperse Ethanol droplets with a median aerodynamic diameter of 1.39 μm. Sample 4# is Silicon dioxide microspheres (Andi Metal Materials Co., Ltd., Hebei, China). Sample 5# is irregular Silicon dioxide particles (Chuangjia Welding Materials Co., Ltd., Hebei, China). Sample 6# is the collected coal ash sample. Samples 7# and 8# are two kinds of Silicon oxide powder materials (Ruilong Biotechnology Co., Ltd., Hebei, China). Sample 9# is Basic magnesium sulfate whiskers (Fengzhu Composite New Material Technology Co., Ltd., Shanghai, China). Sample 10# is self-synthesized rod-shaped Silicon dioxide particles.

**Figure 15:**
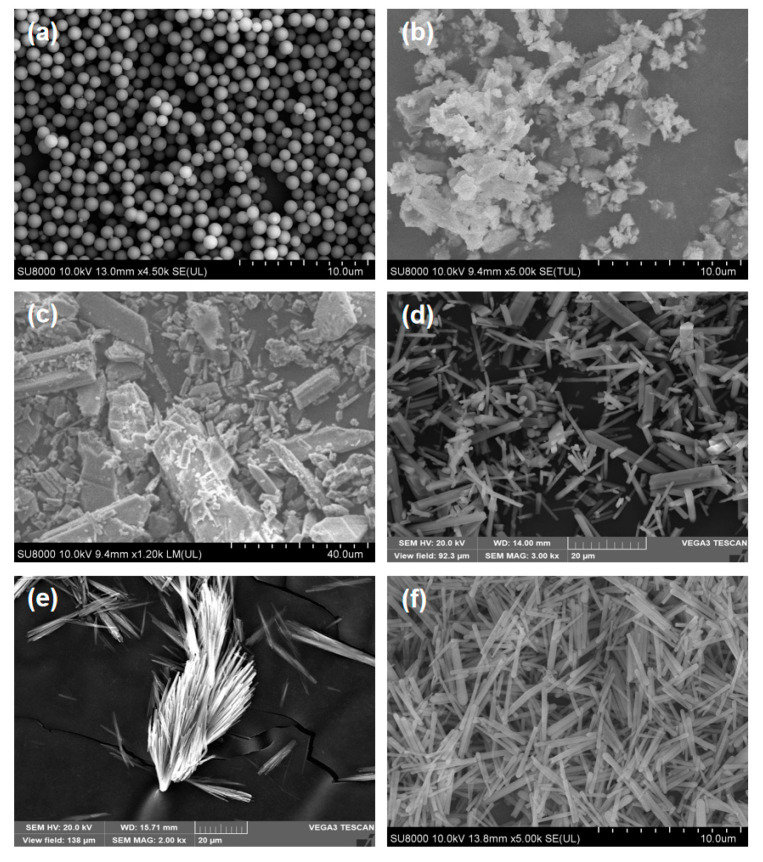
Electron microscope images of aerosol samples. (**a**) 4# Silicon dioxide microspheres. (**b**) 5# irregular Silicon dioxide particles. (**c**) 7# Silicon oxide powder materials. (**d**) 8# Silicon oxide powder materials. (**e**) 9# Basic magnesium sulfate whiskers. (**f**) 10# rod-shaped Silicon dioxide particles.

As can be seen from Figure 14, for the two monodisperse Oleic acid droplets, 95.9% and 99.6% of the particles were identified as spherical, respectively, and 94% of the particles were identified as spherical for monodisperse Ethanol droplets. For coal ash samples, the tested particles were mainly other non-spherical particles (accounting for 47.6%), which is consistent with some research conclusions [33]. In addition, 73.4% of the 4# Silicon dioxide microspheres were identified as spherical, 1.9% as rod-shaped, and 24.7% as other non-spherical particles. More than 48% of the particles in 5# irregular Silicon dioxide particles were identified as other non-spherical particles. For Silicon oxide powder materials, the 7# sample was mainly composed of other non-spherical particles, accounting for 49.9%, and the 8# sample was mainly composed of rod-shaped particles, the corresponding proportion was 53.4%. In the 9# Basic magnesium sulfate whisker sample, rod-shaped particles account for the largest proportion, corresponding to 58.9%. Additionally, in the 10# Silicon dioxide sample, the proportion of rod-shaped particles was as high as 74.1%. Combined with the SEM images of aerosol samples in Figure 15, it can be seen that the classification results of the above aerosol samples are reliable.

## 4. Conclusions

This paper proposed a new method for rapidly identifying and classifying the shape of aerosol particles. Combining the polarized light-scattering and angle-resolved light-scattering measurement technology of individual aerosol particles and based on the multivariate analysis method of particle size, polarized light scattering, and angle-resolved light scattering, a good discrimination effect was obtained for spherical, rod-shaped, and other irregular aerosol particles. Through select the spectral data of scattered light with the same TOF1 (corresponding to aerodynamic diameter), the influence of particle size on the shape classification and the difference of intensity of scattered light from particles was eliminated effectively. The shape recognition and classification model of individual aerosol particles was established by the PLS-DA method, and the ROC curve was applied to analyze the ability to discriminate the shape of aerosol particles. Through nonlinear preprocessing of the spectral information of aerosol particles, the classification effect of the model on the shape of particles is effectively improved. The discriminant analysis of spherical and rod-shaped particles in different aerodynamic particle size ranges shows polarized light scattering and angle-resolved light scattering have their advantages of particle shape recognition in different particle size ranges, and the feasibility of the proposed method for aerosol particle shape discrimination was verified by laboratory experiments.

The rapid identification and classification method of aerosol particle shape proposed in this paper has great significance to supplement other rapid detection methods and improve aerosol particle identification ability. It can provide more information for atmospheric aerosol measurement and has application value in the fields of aerosol particle tracing, atmospheric radiation balance research, respiratory fiber concentration detection, and exposure hazard assessment. To further improve the shape recognition ability of the system, more standard spherical and rod samples (with different refractive indices) will be used for experiments to optimize the particle shape classification model. In addition, to expand the application ability of the proposed technology in various scenarios (such as mobile real-time monitoring of aerosols in a large area based on vehicle or airborne), we also plan to further optimize the mechanical structure, optical path layout, and circuit integration of the apparatus to improve its portability and anti-interference capability.

## Figures and Tables

**Table 1:** The PCTVAR of variables when different numbers of principal components were extracted.

Evaluation Index	ncomp = 1	ncomp = 2	ncomp = 3	ncomp = 4	ncomp = 5
PCTVAR of X	0.3954	0.6165	0.7575	0.8659	0.9131
PCTVAR of Y	0.4506	0.6390	0.6759	0.6864	0.6889

**Table 2:** The results of model evaluation indexes after nonlinear processing.

Evaluation Index	ncomp = 1	ncomp = 2
AUC	0.9825	0.9828
PCTVAR of X	0.7511	0.999
PCTVAR of Y	0.7226	0.724

**Table 3:** Model evaluation indexes obtained by PLS-DA for each particle size segment.

Evaluation Index	D1	D2	D3
AUC	0.9950	0.9905	0.9787
PCTVAR of X	0.9991	0.9990	0.9856
PCTVAR of Y	0.8515	0.7938	0.7029

## Data Availability

The data presented in this study are available on request from the corresponding author.
